# COVID-19: transitioning from in class to online teaching in a heartbeat—Research Methods in Applied Biology

**DOI:** 10.1093/tas/txab014

**Published:** 2021-02-02

**Authors:** Marina A G von Keyserlingk

**Affiliations:** Faculty of Land and Food Systems, The University of British Columbia, Vancouver, Canada

**Keywords:** agriculture, applied biology, safe learning spaces, undergraduate

## Abstract

At the start of the COVID-19 pandemic, Universities around the world were forced to rapidly transition from face-to-face learning environments to online learning. This paper describes this transition through the lens of a professor responsible for a third year Applied Biology class focused on providing undergraduates with research experience. The paper also describes an innovative format used to engage undergraduates in research but also suggests that the creation of safe learning spaces in the virtual world may be key to successful delivery of these types of courses. Lastly, in times of rapid change professors need, on occasion, take a step back and simply listen to their students.

At the University of British Columbia (UBC), Faculty of Land and Food Systems (formerly the Faculty of Agricultural Sciences) I am responsible, together with a colleague and one teaching assistant (TA), for delivering a course—Research Methods in Applied Biology (APBI 398)—intended to provide third and fourth year students with in-depth research experience, and the support and skills that will allow them to succeed in research. This course is taken by students thinking about whether to complete an undergraduate thesis in their final year or simply wanting to know more about how research is done. The course is grounded in experiential learning; each student works with a researcher where they are able to learn research techniques, discuss the research ideas, and help in ways that are useful to the researcher. We have been teaching this course for over a decade using an in-class format that is relaxed, collegial, and supportive of the work and learning that occurs during the mentorship experience. The course is designed for a maximum of 20 students and is given twice a year—in term one (September to December) and in term two (January to April). In normal terms, every Monday the class meets in-person for 3 h.

The course is centered around each student identifying, and collaborating with a scientist working in an area of the student’s interest. During the term, students volunteer for a minimum of 20 h with the scientist, on a current research project. Although most researchers are located on campus, the students are encouraged to venture abroad via the Internet—for example, we have had students working with researchers located in other parts of Canada, Hawaii, California, Falkland Islands, and the United Kingdom. In these cases, the student’s interactions are all virtual. Students are integrated into the research through data entry tasks, video watching, and even attend virtual lab meetings through Skype. Over the years, we have had students working with researchers studying animals, plants, marine biology, zoology, cancer, wildlife conservation, soil science, ecology, and the list goes on.

Briefly, our key learning outcomes are that students, through the lens of the research undertaken in their mentor’s lab, gain an understanding of the main research questions being asked by the researcher and are able to participate firsthand in the research process. To compliment, and showcase their learnings, students are required to give a “news feature” style presentation, designed to be accessible to a general audience. This presentation allows them to develop skills in scientific journalism while also exposing their classmates to different research areas. The students demonstrate understanding of their research area by completing a “scientific conference” style abstract, a poster, and by giving a presentation in a mock conference style setting. The abstract and presentation are based on their work with their mentor and describe their research objectives, methodology, results, and conclusions. Lastly, the capstone requirement for the course is a 10 to 12 page rapid systematic review. We work with them, step-by-step, to summarize and critically evaluate peer-reviewed research papers in their research area. Over the past decade, although not an intended outcome of this class, a number of the students have gone on to complete an undergraduate thesis with their mentor, participate enough in the research to warrant authorship, and even transition to graduate work.

For the 2020 Term 2, things were progressing along as usual, but life took a sharp turn three quarters of the way through the course. Our weekly class on March 9 proceeded as normal: we began with our usual ripple—where each student gave a brief summary of how their research experience had progressed over the past week (including identifying things that have gone well, *or not*). We then discussed what makes a good poster and how a poster presentation differs from an oral presentation. The latter part was achieved in part by the three of us giving poster presentations at a mock conference, with the students circulating the room talking to each of us! At that time, we were all blissfully unaware that this would be the last time we would meet face-to-face. On Friday March 13 at approximately 1:45 p.m., in accordance the Office of the Provincial Medical Health Officer, British Columbia’s Centre for Disease Control and Ministry of Health, UBC canceled all on-campus and off-campus events with more than 250 people. This was followed by a second communication at 5:20 p.m. that same afternoon where it was announced that the entire university was transitioning to online classes effective Monday March 16, 2020 for the remainder of the term.

So here we were about to enter the 11th week of a 13-week term trying to facilitate a course where much of the success of the class is based on being together each week! We use in-class time to check-in with everyone, practice peer-to-peer critical evaluation, and discuss various assignments that the students were required to do during the term. However, at the time of transition to online learning we had not yet completed the peer review of the systematic review, our peer review of the poster, nor our capstone event—the poster presentation. In hindsight, we were lucky regarding the timing of the peer review component of the systematic review, which was set for week 11. As instructors who had never taught online, we were naïve to the different options available for online teaching and were not organized to give the first scheduled class—only 3 days (including the weekend) hence. Thankfully, for the first week where we were online we managed to utilize Canvas—UBC’s online learning platform—that allowed our TA to group students into pairs who could provide “off line” critical feedback to each other on their systematic reviews.

In week 12 (week 2 of online teaching), we embarked on our first Zoom class. It quickly became apparent during this Zoom class that online teaching in the face of a pandemic requires more from us than the traditional “Professor” role as experts—we truly needed to embrace the role of being facilitators. We began the online class with our usual weekly ripple—remember we had all been socially distancing for over 2 weeks which meant that many students had been in social isolation for this period—and it was clear that the students (and arguably us) needed to reestablish our collective sense of community. We needed to create a safe space for all of us to engage with one another, despite communicating through the virtual world. The international students, faced with a unique set of challenges that come with being far from home, needed to hear that they were not alone. All of our students—including those who had moved home to their families—needed to hear that we were flexible and aware that everyone was navigating a unique set of circumstances that clearly challenged the traditional vehicles used for teaching. This first Zoom class, in my opinion, was a huge success because it simply provided everyone the space to chat and support one another. This simple action allowed us to reestablish our community, which is hugely important to our class, and arguably to the success of science.

I am indeed proud of our students, and thankful for their patience with us as we all learned how to transition to an online learning format. Our goal, after loading Zoom onto our computers in our home offices, was to figure out how to “Zoom” in ways that allowed for pedagogical growth. The breakout room tab on Zoom was a life saver as we were able to put the students into small groups of three to four to allow for peer review of their posters. Within each breakout room the students shared their screens one at a time and requested critical feedback from their colleagues. In week 12 (week 3 of online teaching), the students used their recently revised posters to participate in a Zoom poster presentation. Although somewhat less ambitious than originally planned (we had briefly discussed having the entire class participate together), we made the decision to limit the participants in the Zoom call to an individual student and the instructors in 20 min blocks of time. In hindsight, I believe this was the right thing to do given that we were all neophytes on how best to offer such a space, but in the voice of one of students it was clearly a success: “*The online poster presentation was a rare and valuable opportunity to be treated as a scientific equal as an undergraduate*.” (Student, APBI 398, April 2020)

When I look back over the last weeks of our APBI class my lesson learned, in times of challenge such as that experienced by all of us involved in education during the global COVID-19 pandemic, is that it is as important to listen to our students on how their days are going as it is to convey information that they must know in order to fulfill the course requirements. I firmly believe that without the former we will never achieve the latter. We as “Professors” need to constantly remind ourselves that, regardless of whether we are in the same physical room as our students or in a virtual one, we have a privilege and obligation that comes with being facilitators of learning. It is part of our job to encourage dialogue with, and between, our students, including giving time to ask “*how are you today*?” Of course, large class sizes will require innovation to achieve dialogue, but we owe it to our students to establish communities or subcommunities within the learning environment that allows our students to feel supported both from a factual perspective but also as individuals in their learning journey. It is my experience that this supportive environment can be achieved regardless of whether we are face-to-face in the same physical room or online in a virtual classroom. I leave the last words to two of our students about their experiences of transitioning from a face-to-face learning environment to an online learning environment—in what felt like a heartbeat.

“During this time everyone has been adjusting and struggling in their own ways trying to make the best of the situation that they are in. Personally, I am struggling not knowing where I will live in a month or what is happening with my job given the current COVID-19 crisis. I have been looking forward to this course every week because during every meeting you convey to us that you actually care that we are doing okay and that you are available for support, and creating this safe, understanding environment has been a huge necessary stress relief. At UBC, especially in bigger courses, we are often treated like another number in the system, but this class created a community that cares for one another and respects our needs as individuals. I am, forever, grateful to the wonderful professors and the TA for treating us with kindness by providing flexibility and understanding, allowing us to put our best foot forward in our work, especially during these uncertain times.” (Student, APBI 398, April 2020)

“In life, adaptability may be the most relevant virtue to achieve. This course allowed us as students to change how we interact with professors, change the way we understand research methodology, and change the possibilities we see in our future careers. For us, the changes caused by the COVID 19 pandemic were just another obstacle to adapt to. I am grateful that our professors in this class were able to transition the course online smoothly and that all of our projects could be completed in the new format. Throughout this course—before the transition online and after—I have felt supported and encouraged to push my own limits and achieve to my highest potential.” (Student, APBI 398, April 2020)

